# NMR illuminates the pathways to ALS

**DOI:** 10.7554/eLife.08679

**Published:** 2015-06-23

**Authors:** Tao Xie, Charalampos G Kalodimos

**Affiliations:** Center for Integrative Proteomics Research and the Department of Chemistry and Chemical Biology, Rutgers University, Piscataway, United States; Department of Chemistry and Chemical Biology and the Center for Integrative Proteomics Research, Rutgers University, Piscataway, United Statesbabis@rutgers.edu

**Keywords:** human Cu,Zn superoxide dismutase, CPMG relaxation dispersion, non-native oligomers, transient conformations, CEST, protein aggregation, *E. coli*

## Abstract

A combination of NMR techniques is able to explore the structure of short-lived protein conformations.

**Related research article** Sekhar A, Rumfeldt JAO, Broom HR, Doyle CM, Bouvignies G, Meiering EM, Kay LE. 2015. Thermal fluctuations of immature SOD1 lead to separate folding and misfolding pathways. *eLife* **4**:e07296. doi: 10.7554/eLife.07296

**Image** The most unstable form of the enzyme SOD1 is thought to cause ALS

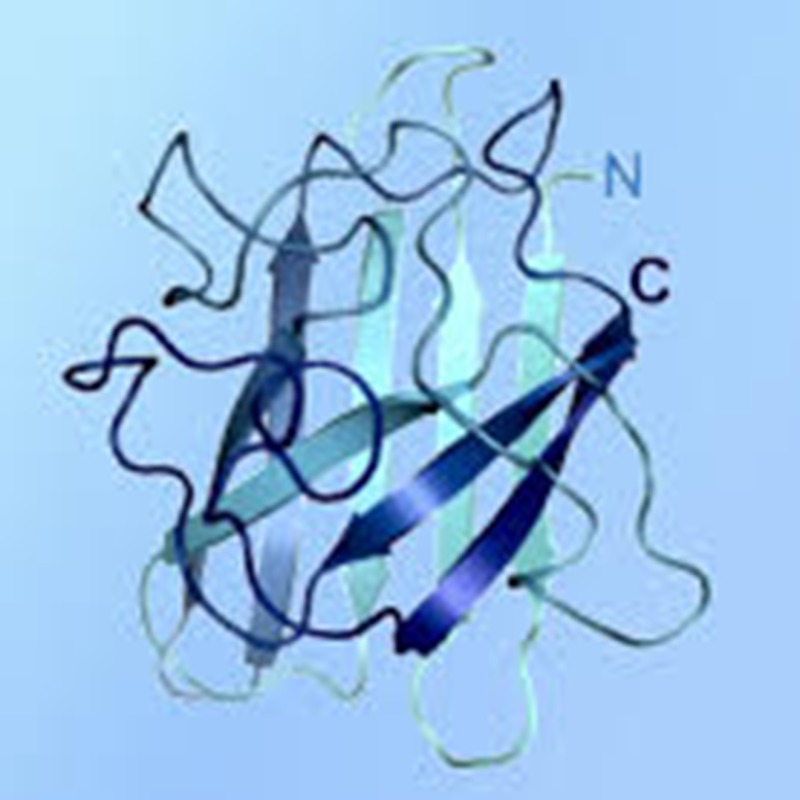

Proteins can fold into many different conformations, and the conformation with the lowest energy is the most stable (Frauenfelder et al., 1991). Two techniques—X-ray crystallography and NMR spectroscopy—are routinely used to work out the structure of the protein in its ground state with atomic resolution. However, a given protein also spends time in other (higher-energy) states, and it has become clear over the past decade that these other states are involved in various biological processes, including protein folding, enzyme catalysis, protein-ligand interactions and protein allostery (Palmer, 2004; Sekhar and Kay, 2013; Tzeng and Kalodimos, 2013). However, as few of the proteins in a sample will be in any of these higher-energy states, most biophysical techniques are not able to detect them.

NMR spin relaxation is a powerful technique that can probe how proteins move over a wide range of timescales, from picoseconds to hours, with atomic resolution (Kay, 2011). Now, in *eLife*, Lewis Kay of the University of Toronto and co-workers—including Ashok Sekhar as first author—have used two complementary NMR spin relaxation methods to explore the different conformations of an enzyme that has been linked to amyotrophic lateral sclerosis (ALS), a devastating neurodegenerative disease (Sekhar et al., 2015).

Mutations in an enzyme called SOD1 have been identified as the main cause of the inherited form of ALS (Rosen et al., 1993). However, little is known about the cause of sporadic ALS, which has the same symptoms but appears to occur randomly throughout the population. The detailed molecular mechanisms of ALS remain to be clarified, although the inherited and sporadic forms of the disease are thought to share a common pathway. The detection of insoluble SOD1 in ALS patients suggests that mutations and modifications could lead to conformational changes in SOD1 that, in turn, increase the chances that it will misfold and form insoluble aggregates (Bosco et al., 2010).

The SOD1 enzyme can take on many different structural forms. The active form of the enzyme, known as Cu_2_Zn_2_SOD1^S-S^, exists as a dimer made up of two identical subunits. Each subunit is a β-barrel with eight strands, the most important of which are called the Zn loop and the electrostatic loop. Each subunit also contains a zinc (Zn) ion and a copper (Cu) ion. The Zn loop serves as the binding site for the Zn ion, while the electrostatic loop stabilizes the binding of both ions. There is also an intramolecular disulfide bond that further stabilizes the structure by anchoring the Zn loop to the β-barrel.

In contrast, the most immature and unstable form of the enzyme, apoSOD1^2SH^, exists as a monomer and does not contain any metal ions or disulfide bonds. This form of the enzyme is thought to be the toxic species that causes ALS (Rotunno and Bosco, 2013), so there is a clear need to learn more about its structure and other properties. Sekhar et al.—who are at the University of Toronto and the University of Waterloo—found that the Zn and electrostatic loops were much more flexible in apoSOD1^2SH^ than in Cu_2_Zn_2_SOD1^S-S^. However, the β-barrel structures of both forms are very similar.

If a molecule switches between its ground and excited states around 200–2000 times per second, and more than ∼0.5% of the sample is in the excited state at any one time, a form of NMR called CPMG relaxation dispersion provides detailed information about the excited states (Sekhar and Kay, 2013). A different NMR technique called CEST can be used when the molecules switches between conformations around 20–300 times per second (Fawzi et al., 2011; Vallurupalli et al., 2012). Sekhar et al. demonstrated that a combination of CPMG and CEST can elucidate multiple exchange processes between different conformations. Moreover, despite the underlying complexity of the processes, they were able to determine the thermodynamic, kinetic and structural properties of four short-lived excited states that are in equilibrium with the ground state of apoSOD1^2SH^ (Figure 1). Measurements of chemical shift differences indicated that two of the excited states were actually native conformations of the Cu_2_Zn_2_SOD1^S-S^ dimer (Figure 1A).

**Figure 1.**
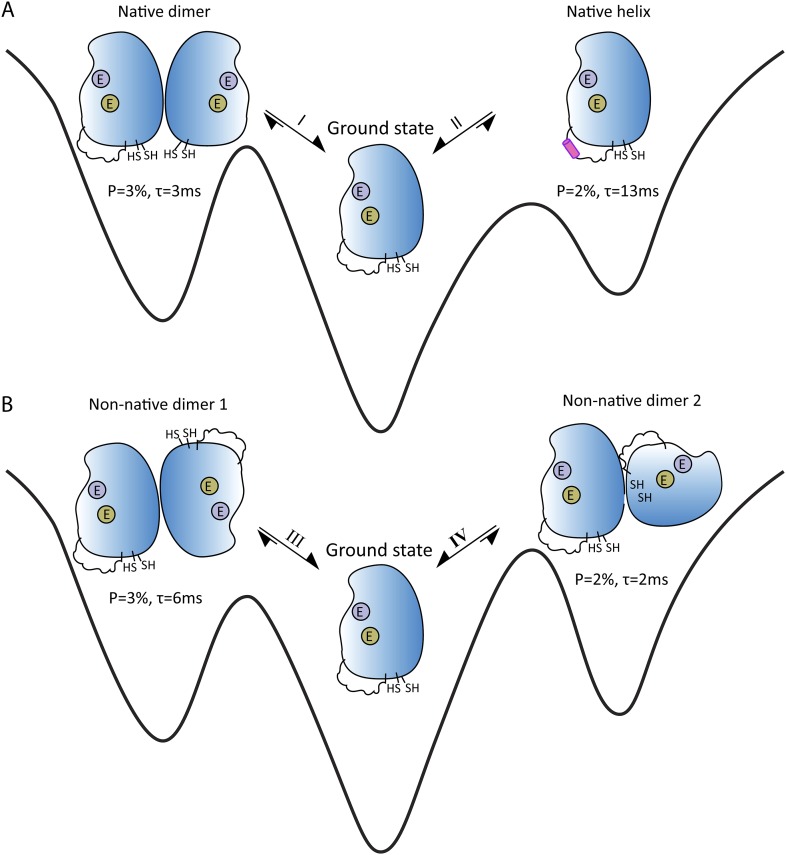
Energy landscape showing the four short-lived excited states that are in equilibrium with the ground state of apoSOD1^2SH^, which is thought to be the form of the SOD1 enzyme that causes ALS. (**A**) The ground state (center) is in equilibrium with two native (or working) conformations. Exchange process I leads to the formation of a dimer, with the changes being localized to the surface that forms the interface between the two SOD1 monomers in Cu_2_Zn_2_SOD1^S-S^ (left); exchange process II folds the electrostatic loop within the enzyme to form a helix (pink). (**B**) The ground state (center) is also in equilibrium with two non-native conformations, both of which have aberrant dimer interfaces. These interfaces and the unstructured electrostatic loop in apoSOD1^2SH^ may act as sites for the formation of higher-order oligomers and aggregates that may have a role in ALS. The binding sites for metal ions are denoted by purple circles (Zn) and khaki circles (Cu); these sites are empty (denoted by E) for all these states. *P* is the percentage of enzymes in a state; τ is the lifetime of the state.

Of particular interest are the conformational exchanges between apoSOD1^2SH^ and two non-native oligomers (Figure 1B). If factors such as mutations or modifications shift the equilibrium towards these two states, they might serve as starting points for the formation of more complex oligomers that could have a role in ALS.

By showing how NMR spin relaxation methods can reveal such details, the approach developed by Sekhar, Kay and co-workers has the potential to assist in the design of therapeutic molecules that target these oligomers.
